# A cross-lingual analysis of attitudinal meaning in publicity discourses of Anglo-American and Chinese universities: a psychological insight from the appraisal system

**DOI:** 10.3389/fpsyg.2025.1635204

**Published:** 2025-10-10

**Authors:** Lu Zheng

**Affiliations:** School of Humanities, Jilin University, Changchun, China

**Keywords:** attitudinal meaning, publicity discourse, appraisal system, affect, judgement, appreciation, cross-cultural communication

## Abstract

**Introduction:**

University publicity discourse is pivotal to global communication, embodying institutional values and cultural identities. While linguistic research on its embedded attitudinal meaning abounds, cross-lingual (English-Chinese) comparative exploration—especially systematic analysis of attitudinal resources in such discourses—remains limited, forming the core focus of this study.

**Methods:**

Drawing on the Appraisal System (a robust framework in Systemic Functional Linguistics), this research conducted a comparative analysis of English and Chinese university publicity discourse samples. It extracted and categorized attitudinal resources (Affect, Judgment, Appreciation) and compared their distribution and expressive differences via qualitative and quantitative methods.

**Results:**

Significant cross-lingual disparities emerged: English discourse used richer emotional expressions (Affect/Appreciation) to enhance affinity, while Chinese discourse prioritized objectivity and authority with restrained emotions. No notable differences were found in Judgment resources. English discourse emphasized moral constraints, whereas Chinese discourse focused more on legal constraints.

**Discussion:**

This study uncovers distinct attitudinal meaning patterns across cultural-linguistic contexts, filling cross-lingual research gaps and refining the Appraisal System. Beyond linguistic theory, it offers practical guidance for universities to optimize cross-cultural publicity and boost global communication effectiveness.

## 1 Introduction

In the context of the continuous advancement of higher education within the Chinese educational landscape, the construction of the “Double First-Class” initiative—encompassing the cultivation of world - class universities and first - class disciplines—is being propelled forward with substantial momentum (Zhang et al., 2023). To materialize this strategic objective, institutions of higher learning are unremittingly dedicated to the renewal of educational ideologies, the recruitment of preeminent faculty members, the reinforcement of collaborative endeavors with other academic and non - academic entities, and the facilitation of the fruition of scientific and technological research undertakings (Guo, 2020). These accomplishments can be efficaciously promulgated both domestically and on the international stage through the medium of publicity discourses.

The British translation theorist Newmark (1988) was the first to put forward the concept of publicity discourse. When studying discourse translation, from a functional perspective, he classified discourses into three categories, namely: informative discourse, expressive discourse, and vocative discourse. Publicity discourse is classified as vocative discourse. Publicity discourse is of great significance to study because it serves as an important means of publicity and a window for communication. There are various types of publicity discourse, which are applied in corresponding fields, such as society, urban development, economy, culture, and education, etc. (Newmark, 1988). Due to the multi-type characteristics of publicity discourse, multi-dimensional studies related to social, political, economic, cultural, and educational issues are carried out (Danie and Amodeo, 2014; Serensen, 2015; Kogen, 2015; Brito et al., 2017; Beals, 2017; Wang and Zhang, 2019; Ding, 2022; Le and Ngai, 2022; Li and Tang, 2022).

In terms of social, political and educational issues, studies on publicity discourse have explored its dynamic interaction with real-world changes from multiple angles. Danie and Amodeo (2014), through analyzing rural publicity advertisements in the magazine Global Rural from 1980 to 2010, pointed out that changes in advertising publicity discourses reflect the diversification of rural identity and social changes. Similarly, (Kogen, 2015) evaluated the role of celebrity effect in publicizing global humanitarian crises using critical discourse analysis, highlighting how such discourse shapes public perceptions during critical events. Brito et al. (2017), on the other hand, shifted focus to educational institutions, exploring the discursive functions of English on the official websites of private foreign language colleges and finding that despite their corporate nature, these institutions tend to use educational and teaching-oriented publicity.

While social-oriented studies emphasize the reflection of publicity discourse in tangible social shifts, research on publicity discourse and culture delves deeper into its role in cross-cultural communication and ideological transmission. Serensen (2015), integrating Habermas’ thoughts on Bildung, explored the functions of university publicity discourse from the perspectives of international publicity, discourse, and politics, revealing its connection with educational philosophy and social ethics. Based on Nida’s cultural classification, Ding (2022) analyzed the cultural translation methods for Korean-Chinese publicity discourse and the strategies for introducing Chinese cultural elements into Korean, providing practical insights for cross-linguistic cultural communication. Li and Tang (2022) further studied urban publicity discourse, emphasizing that translation needs to adapt to the habits of the target language, overcome cultural differences, and analyzed the issue of cultural vacancies, thus enriching the understanding of urban image construction in intercultural contexts. Beyond social and cultural dimensions, Beals (2017) expanded the research horizon by exploring the intersection of publicity discourse and art. Against the background of Dadaism, he found that its advertising discourse can participate in debates between art and advertising, providing a new perspective for publicity research that bridges aesthetic expression and communication purposes.

Collectively, these studies have analyzed the multifaceted connections and mutual infiltration between publicity discourse and the macro world, highlighting its diverse roles in the fields of society, education, culture and so on. While differing in research objects and methodologies, they collectively underscore the complexity of publicity discourse as a practice in the context of society, education, culture, etc. However, the scholars from different fields have diverse understandings of publicity discourse, resulting in rather fragmented research on it. The common feature of all types of publicity discourse is external communication, and the emotions, judgments, and values expressed in the discourse play a crucial role in successful communication. However, these aspects are less explored in existing studies.

All these aspects are reflected in the attitudinal meaning of the publicity discourse (Martin and Rose, 2003). The attitudinal meaning can be realized by Attitudinal System, which is a core part of Appraisal System developed by J. R. Martin, a functional linguist. As far as the analytical modes of attitudinal meaning are concerned, “the study of attitudinal meaning in discourse analysis can be roughly categorized into two groups according to how the attitudinal meaning is analyzed” (Song, 2015, pp. 383–405). One is structural analysis, which considers attitudinal meaning as an integral component. The attitudinal meaning should be generated through interaction with other components. The other is stratified analysis, which analyzes attitudinal meaning in two steps. In the first step, the attitudinal meaning is identified and categorized at the lexical level. In the second step, the discourse is analyzed based on the attitudinal lexis. For the current research purpose, we conduct a stratified analysis based on the Appraisal System. Through the analysis, this study not only reveals the unique patterns through which attitudinal meaning is realized across diverse cultural and linguistic landscapes but also enhances the Appraisal System framework via domain-specific case analyses. By narrowing the divide between theoretical constructs and cross-lingual practical applications, it enriches the theoretical arsenal of Systemic Functional Linguistics and presents innovative angles for cross-cultural discourse research. Beyond its contributions to linguistic theory, the findings offer tangible guidance for fostering effective cross-cultural communication in university publicity contexts.

## 2 Literature review

### 2.1 Attitudinal meaning and appraisal system

In the early 1990s, Martin began to study the Appraisal System (also known as Appraisal Theory). The Appraisal System is an update and development of the interpersonal meaning in Systemic Functional Linguistics, providing a powerful theoretical foundation for the study of the interpersonal meaning of discourses. “Appraisal is a system of interpersonal meaning” (Martin and Rose, 2003, pp. 26). Meanwhile, “Appraisal is related to the evaluation of values. Attitude is constructed within the text, which involves the intensity of emotions, the way values are distributed, and compatibility with the readers” (Martin and Rose, 2003, pp. 25). In brief, “the Appraisal System is a complete set of resources for expressing attitudes through language” (Wang and Ma, 2007, pp. 20). Although the Appraisal System has only been in existence for about 30 years, it has attracted great interest from the academic community, and they have conducted various studies based on it. Among them, Wang (2001) first introduced the Appraisal System into China, while Li (2001, 2004) was the first to apply the Appraisal System to discourse practice.

The Appraisal System consists of three major systems: Attitude, Engagement, and Graduation. In the Appraisal System, a system is a resource, and each subsystem is a kind of resource for analyzing the interpersonal meaning of discourses (Martin and White, 2005). Among them, the Attitudinal System is the core system of the Appraisal System. “Attitude refers to the judgments and appreciations made of human behaviors, texts/processes, and phenomena after being influenced psychologically” (Wang and Ma, 2007, pp. 20), which includes three subsystems: Affect, Judgment, and Appreciation.

**Figure 1:**
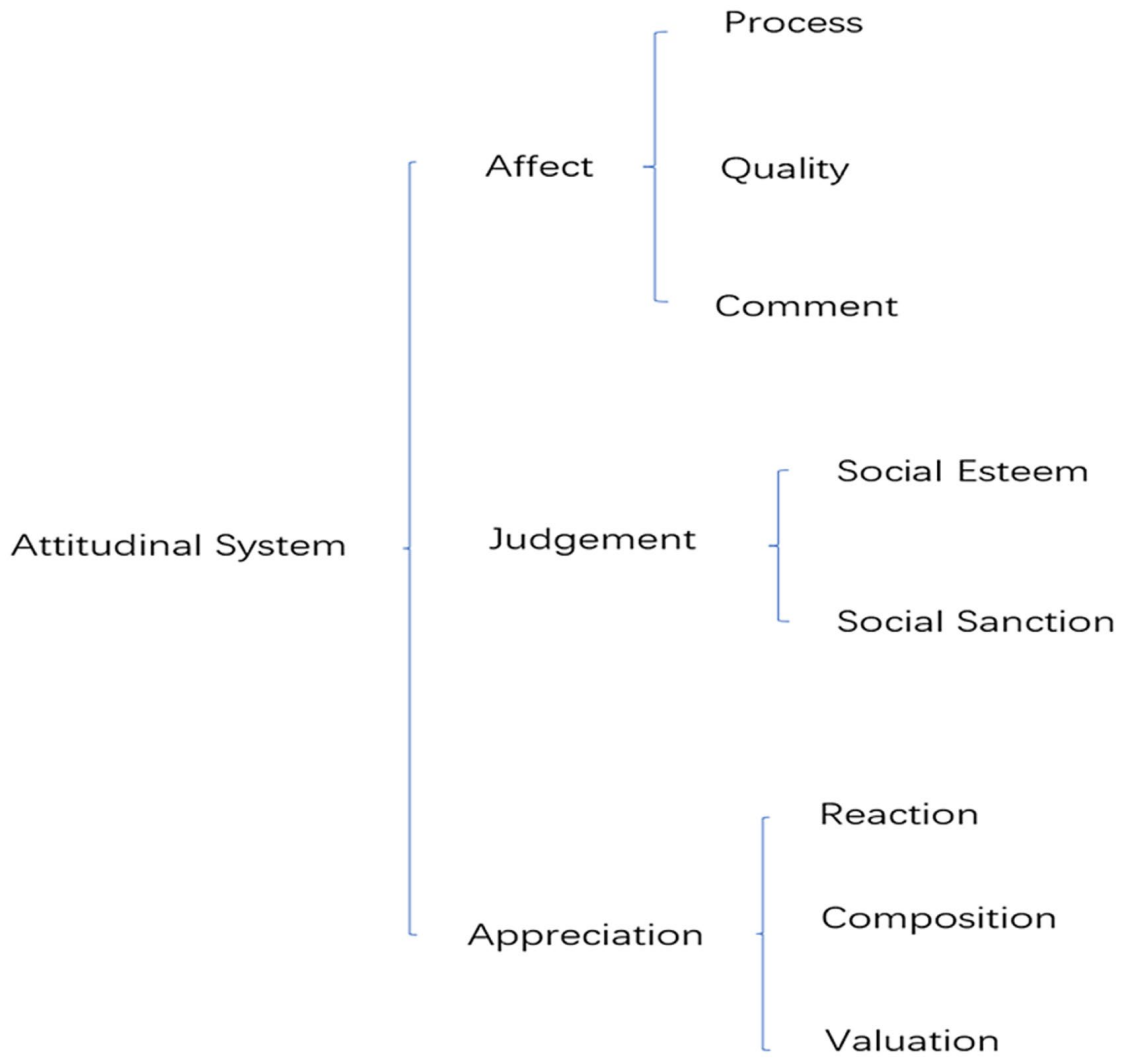
Attitudinal system (Based on Appraisal Theory).

From a psychological perspective, the Affect System represents the emotional responses to behaviors and phenomena; it can be further divided into three types: Quality, Process, and Comment (Martin and White, 2005). The Judgment System, from an ethical perspective, conducts moral evaluations of the behaviors of language users (Martin and White, 2005). “As a resource for explaining language phenomena, the judgment system is used to explain the moral judgments made by language users on a certain behavior according to ethics/morals (rules and regulation)” (Wang, 2001). It is divided into two parts: Social Sanction and Social Esteem. The Appreciation System is the evaluation of objects and products from an aesthetic perspective (Martin and White, 2005). According to Wang (2001), appreciation system, as a resource to explain linguistic phenomena, is used to explain language users’ appreciation of the aesthetic character of texts/processes and phenomena. It includes three parts: Reaction, Composition and Valuation. The schematic representation of Attitudinal System is illustrated in Figure 1.

However, according to the different types of discourses to be analyzed, the objects of “Judgment” and “Appreciation” are different. The object of Judgment is not necessarily a person, and the object of Appreciation is not necessarily a thing (Martin, 1992). For example, in the publicity discourses of universities analyzed in this study, the university itself, as a participant who initiates actions, can be the object of Judgment. Similarly, the students cultivated by the university and the faculty members recruited can be regarded as the “products” of the university and analyzed as the objects of Appreciation.

### 2.2 The studies on attitudinal meaning

Since the birth of the Appraisal System, the study of attitudinal meaning in various types of discourses has always been a subject of great concern. Numerous scholars have explored it from different perspectives and using a variety of methods, gradually constructing a rich and diverse research landscape.

In recent years, scholars have started to focus on the analysis of attitudinal meaning in specific discourse types. For instance, Song (2015) selected 15 English short stories to construct a small-scale corpus, delving deeply into the lexical realization of attitudinal meaning and its coding process in discourse analysis. Through meticulous research, Song proposed the distinction between the typical realization and the combinational realization of attitudinal meaning, and further elaborated on the differences between independent and correlational realization, as well as projected and projecting realization. This has opened up a new path for subsequent exploration of attitudinal meaning at the lexical level, enabling researchers to have a clearer understanding of the micro-level manifestation forms of attitudinal meaning in discourses.

As the research has advanced, the scope has been continuously expanded to the macro-level interpretation of literary works. Leng (2017), based on the Attitudinal System of the Appraisal Theory, turned his attention to the classic novel Gone with the Wind. By means of a combination of qualitative and quantitative analysis methods, Leng thoroughly explored the distribution and proportion of attitudinal resources in the novel’s text. This research not only helps in deeply understanding the attitudes conveyed by the author in the work but also lays a solid foundation for the subsequent comparative analysis of attitudinal meaning in translation studies.

At the same time, news discourse, as an important carrier of information dissemination, has also become a key area for studying attitudinal meaning. Li (2024b) conducted an in-depth analysis of the news reports in China Daily regarding the discharge of nuclear-contaminated water from Fukushima. It was found that the use of affect resources was the most frequent, followed by judgment resources and appreciation resources in sequence, and the overall ecological tendency was mainly destructive. This research result helps readers to see through the surface of news texts and gain insights into the hidden ideology and ecological thoughts behind them, enhancing the public’s ability to deeply interpret news discourse. In the same year (2024), Li also explored the news discourse about the COVID-19 pandemic in China Daily. Using quantitative and qualitative research methods, it was revealed that judgment resources appeared most frequently, while affect and appreciation resources accounted for a relatively small proportion. In terms of the overall attitudinal polarity, positive attitudes outweighed negative attitudes. This series of studies on news discourse has shown us the diverse manifestation forms of attitudinal meaning in news reports on different topics and the possible communication intentions behind them.

In the aspects of new media and the development of young people’s consciousness, Guslyakova et al. (2020) carried out relevant research. They focused on analyzing the phenomenon of psychological attitudes and their impacts on the development of young people’s consciousness and worldviews. By means of methods such as correlation, clustering, and factor analysis, the research revealed various positive correlations between young people’s interaction with new media discourse and their attitudes toward different events, people, and news, providing empirical evidence for understanding the mechanisms of attitude formation and transformation among young people in the new media era. Meanwhile, Telesiene and Hadler (2023), by using systematic literature review and quantitative content analysis, deeply explored the historical stages, theoretical diversity, and empirical evidence of the academic discourse on environmental attitudes and behaviors, sorting out the development context of this academic field and providing a macroscopic historical perspective and theoretical framework reference for subsequent related research. Besides, there are also other scholars conducted related studies on news or media discourses from various perspectives (Buure et al., 2024; Zeng and Zhu, 2024; Wu et al., 2025).

In some special fields, such as courtroom discourse and product introduction discourse, scholars have also conducted in-depth explorations. Shi (2018) analyzed the attitudinal expressions in the audio transcripts of eight court trials and believed that judgment was the most main way for courtroom participants to express attitudes, while the frequencies of using appreciation and affect were relatively low. Moreover, significant differences in attitudinal expressions among all parties in different types of court trials were found. This research helps us understand the attitude construction and power game in courtroom discourse. Deng (2023) conducted a comparative analysis of the English online introductions of men’s and women’s cosmetics and found that when facing female customers, the author tended to use more objective expressions and made more use of appreciation resources to describe product features; while the introductions of men’s cosmetics adopted more diverse ways to directly attract potential customers. This reveals the presentation strategies of attitudinal meaning in product introduction discourse for different audiences.

From a comprehensive view of these studies, scholars have used diverse research methods to deeply explore the manifestation, distribution, and influence of attitudinal meaning in different discourse types, providing us with rich perspectives for understanding the relationship between language and attitudinal expression. However, there are still some deficiencies in the existing research. Most of the studies are limited to a single language environment, and there are relatively few comprehensive cross-lingual comparative studies. Under different language and cultural backgrounds, cultural and social factors profoundly shape attitudinal expressions, and there may be significant differences in the expression and understanding of attitudinal meaning. For example, in the current development of higher education, the differences in value orientations and thinking patterns between Chinese and English cultures will be reflected in the application of attitudinal resources in the publicity discourses of Chinese and foreign universities. The lack of such comprehensive research restricts our comprehensive understanding of the universality and particularity of attitudinal meaning.

In view of this, this study takes the publicity discourses selected from the official websites of Chinese and Anglo-American universities as the corpus and uses the Attitudinal System of the Appraisal Theory as the theoretical framework to conduct a comparative study of Chinese and English publicity discourses, revealing the differences and similarities in the application of attitudinal resources in Chinese and English publicity discourses, facilitating cross-cultural communication and improving the communication effect. Thus, it aims to solve the following problem: How do the attitudinal meaning in the publicity discourses of Chinese universities differ from that in the publicity discourses of Anglo-American universities?

## 3 Methodology

### 3.1 Corpus

University profiles act as quintessential publicity discourses for universities, effectively mirroring their roles in external promotion. In this study, the profiles of 40 Chinese “Double First-Class” universities (universities with world-class universities and disciplines) and 40 British and American universities ranked among the top 500 in the QS World University Rankings (2022 edition) have been meticulously selected from their official websites. These selected profiles are then used to construct Chinese and English corpora for the purpose of comparative analysis. All the universities chosen in this study are renowned educational institutions, covering a wide range of academic fields such as liberal arts, science, engineering, and comprehensive disciplines. This diversity ensures that the university profiles are highly representative of similar institutions within their respective educational landscapes. Specifically, the Chinese corpus is composed of 2,976 sentences and contains a total of 84,832 Chinese characters. On the other hand, the English corpus consists of 1,232 sentences and has 18,828 words. This quantitative information about the corpora provides a solid foundation for the subsequent in-depth analysis of the attitudinal resources and other linguistic features within the university profiles.

### 3.2 Comparability

According to Halliday’s “register theory” (Halliday and Hasan, 1985), language exhibits various forms, known as functional varieties of language, which arise due to changes in situational context. Register encompasses three dimensions: field, tenor, and mode. Field pertains to the communicative theme of discourse. Tenor refers to the social relations and communicative purposes between interacting parties. Mode denotes the channels or media employed in language communication, such as written language or colloquial style. In terms of field, both English and Chinese discourses serve as university profiles, providing an overview of the institution and facilitating external communication. Concerning tenor, they both aim to establish effective communication between university officials and readers in order to achieve their communicative goals. From the mode perspective, both employ written forms with dialogic implications. They share the same genre, subject matter, communicative intention, and corresponding linguistic structure. Consequently, the selected English and Chinese discourses are comparable. Furthermore, in terms of content structure, English and Chinese discourses comprise topics such as university property, development history, faculty, facilities, scientific research achievements, awards, and more. Henceforth, the content structure exhibits similarities. Regarding quantity, Chinese discourses tend to be extensive and detailed while English discourses adopt a concise approach with a more general language style. However, to mitigate the issue of length disparity between English and Chinese discourses during data analysis, ANOVA and frequency analysis techniques are employed, consequently ensuring comparability of the selected corpus from a quantitative perspective. Building upon this foundation, further processing of the data is conducted in the subsequent section.

### 3.3 Data analysis

Firstly, this study established a standardized annotation framework for attitudinal resources to ensure the objectivity and reliability of data extraction. Specifically, we derive the definitions and samples of attitudinal resources from classical literature (Wang, 2001; Martin and Rose, 2003; Martin and White, 2005; Wang and Ma, 2007) (see Table 1). Based on these definitions and samples, we proceed to identify and quantify the attitudinal resources present in the corpus. Two trained annotators (both with a master’s degree in applied linguistics and prior experience in discourse analysis using the Appraisal System) independently participated in the corpus annotation. The annotation process was divided into two phases:

**Table 1:** Definitions and samples of attitudinal resources.

Attitudinal Resources	*Affect*	Meaning	From the perspective of psychology, affect represents the emotional response to behaviors and phenomena. It can be divided into three aspects: process, quality, and comment. Process affect refers to the evaluation of the psychological process of the person evaluated; quality affect refers to the evaluation of the behavior and attributes of the person evaluated; comment affect refers to the overall emotional evaluation to reflect the emotional experience.
		Sample	Nouns, verbs, adjectives, and adverbs, such as: happy, ecstatic, proudly, love, hate, proud; 快乐的 (happy), 满意的 (satisfied), 幸运地 (luckily), 感激(appreciate), etc.
		Example	He is a happy boy. We were ecstatic. 我们的老师是一个快乐的人 (Our teacher is a happy person). 她是很满意的 (she is very satisfied). (quality affect) I love her and also hate her. 我很感激她 (I appreciate him a lot). (process affect) Proudly, the newly established company introduced to the world its first coin counting machine. 非常幸运, 我一次性通过了考试 (Luckily, I passed the exam on my first try). (comment affect)
	*Judgement*	Meaning	Judgment is divided into two parts: social esteem and social sanction and it carries out evaluation on the behavior of language users from the perspective of ethics. Social esteem is used to evaluate whether a person is talented, perseverant, honest and so on. Social sanction is used to evaluate whether a person’s behavior is justified, whether it conforms to the rules and regulations, whether it is true and reliable, etc.
		Sample	Nouns, verbs, adjectives, adverbs, such as: equivalency, praise, guilty, innocent, respectable; 有罪的 (guilty), 有才的 (talented), 诚实的 (honest), 赞扬 (praise), etc.
		Example	respectable members of their communities; 她是一个有才的人(She is a talented woman). (social esteem) He is innocent. 他是有罪的 (He is guilty).(social sanction)
	*Appreciation*	Meaning	Appreciation is the evaluation of objects and products from the perspective of aesthetics, including reaction, composition and valuation. Reaction refers to people’s reaction to the product, such as: whether the person is enthusiastic, whether the product is famous; composition refers to whether the internal structure of the product is balanced, whether it has characteristics, whether it is harmonious; valuation refers to whether the product has practicability, whether it has value, etc.
		Sample	Nouns, verbs, adjectives, adverbs, such as: valuable, well - built, enormously, strong, great response; 匀称的 (well-proportioned), 坚固的 (sturdy), 著名的 (famous), etc.
		Example	The founders provided the most valuable and well-built machines. 她买了一栋匀称且坚固的房子 (She bought a well-proportioned and sturdy house). (valuation, composition) “Positive and negative ion collider” developed by our university produced great response. 鲁迅写了很多部著名的小说 (Luxun wrote lots of famous novels). (Reaction)

Pilot annotation: a subset of the corpus (10% of the total, i.e., 4 Chinese university profiles and 4 Anglo-American university profiles) was selected for pilot annotation. After annotation, the two annotators compared results, discussed discrepancies (e.g., whether “renowned” in English should be categorized as “valuation” or “reaction”), and revised the annotation manual to resolve ambiguous criteria—ensuring consistent understanding of core concepts.

Formal annotation: the remaining 90% of the corpus was annotated independently by the two annotators. After completing formal annotation, inter-annotator agreement was calculated using Krippendorff’s alpha (a widely used indicator for multi-annotator reliability), yielding a coefficient of 0.87. This value exceeds the generally accepted threshold of 0.80 in linguistic research, confirming that the annotation results were sufficiently reliable and minimizing subjective bias in identifying attitudinal features.

Subsequently, an ANOVA (Analysis of Variance) analysis is carried out by means of Excel 2019 to explore the disparities in attitudinal resources between English and Chinese publicity discourses. Employing ANOVA in Excel enables a scientific assessment of the significance of differences between the two sets of data, with *p*-value serving as an indicator for determining both the presence and magnitude of such differences (Park et al., 2009; Rasch and Verdooren, 2020). Previous studies have utilized ANOVA to examine significant disparities within diverse research domains (Park et al., 2009; Kim, 2022). This analysis centers on the *F* value, the F critical (F crit) value, the *p*-value and η^2^ value for different variables within both discourses. In detail, the F value is calculated as the ratio of the mean square (MS) between groups to the mean square within groups. A higher value of this ratio implies more substantial differences existing between the groups. The F crit value stands for the critical F value at a pre-specified significance level. Meanwhile, the p-value reflects the likelihood associated with the observed F value. η^2^ is the effect size, referring to partial eta squared and η^2^ = SS_net_ (sum of squares between groups)/SS_total_ (total sum of squares). To assess the statistical significance, we rely on the following criteria for evaluating these values: When the F value surpasses the F crit value, it serves as an indication of a significant difference. If the *p*-value is less than 0.01, the difference is regarded as extremely significant; when the *p*-value ranges between 0.01 and 0.05, the difference is considered significant; and in the case where the F value is lower than the F crit value or the *p*-value exceeds 0.05, it suggests that there is no significant difference. η^2^ value exceeds or equals 0.06, standing for medium to large effect; η^2^ value exceeds or equals 0.01, standing for measurable effect; η^2^ value is less than 0.01, standing for negligible effect.

When a particular resource demonstrates either an extremely significant or a significant difference between English and Chinese discourses, we proceed with a more in-depth analysis of its usage frequency. This step aims to determine in which of the two discourses the resource is more commonly used. Based on the outcomes of these analytical processes, we elaborate and discuss the similarities and differences that exist between English and Chinese discourses.

This comparative research combining quantitative and qualitative methods enables a more thorough understanding of the characteristics and functions of attitudinal resources in English and Chinese publicity discourses, facilitating a more insightful exploration of the linguistic and communicative differences between the two languages in the context of university publicity.

## 4 Results and discussion

In this section, we first conduct an overall analysis, and then affect, judgement and appreciation resources are analyzed in detail, respectively.

### 4.1 Overall analysis of attitudinal resources

According to the Appraisal System, the object of judgment pertains to the person (the subject of behavior), while that of appreciation is the thing (the product). Nevertheless, considering the characteristics of university publicity discourse, the university itself, which is the subject of behavior within this discourse, is treated as the object of judgment. Its achievements, outstanding students, and technological products are regarded as the objects of appreciation. Moreover, the emotion conveyed in the discourse is considered as the object of affect. The overall frequency of attitudinal resources is presented in Table 2.

**Table 2:** Quantity and frequency of attitudinal resources.

Discourse types	Attitudinal resources	Affect resources	Judgement resources	Appreciation resources
English discourse	472	44 (9.3%)	264 (66.7%)	164 (34.8%)
Chinese discourse	701	12 (1.7%)	364 (51.9%)	325 (46.4%)

The values are rounded to one decimal place.

The subsequent content conducts a differential analysis of attitudinal resources in the publicity discourses of Anglo-American and Chinese universities from the perspectives of affect, judgment, and appreciation (The columns in the table show the comparative data between English and Chinese attitudinal resources, which are the key data indicators of this paper, and the same below). This analysis aims to discern the extent of variation in the utilization of attitudinal resources.

The findings presented in Column 1 of Table 3 regarding affect resources reveal that there is an extremely significant difference between English and Chinese discourses. This is evidenced by the fact that the *F* value is 12.1165, while the F critical value is 4.091279, with F being greater than F crit, the *p*-value standing at 0.001247, which is less than 0.01, and η^2^ value is 0.1221, which is greater than 0.06. Additionally, Table 2 illustrates the frequencies of affect resources in English and Chinese discourses, which are 9.3 and 1.7%, respectively. Evidently, the frequency of affect resources in English discourse is higher than that in Chinese discourse. Consequently, English discourse places a greater emphasis on emotional expression compared to Chinese discourse. It endeavors to engage readers by employing an approachable, enthusiastic, and positive language style. In contrast, Chinese discourse places a greater premium on objectivity and authority, thereby leaving readers with an impression of formality and professionalism.

**Table 3:** Difference analysis of attitudinal resources.

Source	SS	df	MS	*F*	*p*-value	F crit	η^2^
Row	50.8	39	1.302564	1.23301	0.258083	1.704465	
Column 1	12.8	1	12.8	12.1165	0.001247	4.091279	0.1221
Deviation	41.2	39	1.05641				
Amount	104.8	79					
Row	1098.2	39	28.15897	1.22158	0.267482	1.704465	
Column 2	16.9	1	125	4.022692	0.055152	4.091279	0.0079
Deviation	1007.1	39	23.05128				
Amount	2122.2	79					
Row	665.4875	39	17.06378	0.757538	0.805148	1.704465	
Column 3	324.0125	1	324.0125	14.38437	0.000506	4.091279	0.1734
Deviation	878.4875	39	22.52532				
Amount	1867.988	79					

Moreover, the outcomes of Column 2 in Table 3 for judgment resources indicate that the difference between English and Chinese discourses is negligible. Specifically, the *F* value is 4.022692, the F critical value is 4.091279, with F being less than F crit, the *p*-value is 0.055152, which exceeds 0.05, and η^2^ value is 0.0079, which is less than 0.01. Therefore, both English and Chinese publicity discourses accord significant importance to the behavior of the agent (the school) and its pivotal role in the development of the institution.

Furthermore, the results of Column 3 in Table 3 for appreciation resources demonstrate that there is an extremely significant difference between English and Chinese discourses. The *F* value is 14.38437, the F critical value is 4.091279, with F being greater than F crit, the *p*-value is 0.000506, which is less than 0.01, and η^2^ value is 0.1734, which is greater than 0.06. Meanwhile, as shown in Table 2, the frequencies of appreciation resources in English and Chinese discourses are 34.8 and 46.4%, respectively. The frequency of appreciation resources in Chinese discourse is higher than that in English discourse. From these results, it can be inferred that Chinese discourse allocates more textual space to promoting the university’s talent cultivation capabilities, scientific research prowess, and academic accomplishments. The English and Chinese publicity discourses exhibit distinct characteristics in the utilization of the aforementioned three types of resources, and these aspects will be analyzed in greater detail in the subsequent sections.

### 4.2 Affect resources

Martin and Rose (2003) have defined the resources utilized for expressing emotions within a discourse as affect resources. Affect encompasses both positive and negative emotions. Positive affect elicits a sense of pleasure and contentment, whereas negative affect instills feelings of pessimism and despondency. Considering the unique characteristics of publicity discourse, both English and Chinese discourses are found to convey only positive emotions. Affect resources can be categorized into three aspects: process, quality, and comment. Table 4 presents the quantity and frequency distributions of these affect resources, which are as follows:

**Table 4:** Quantity and frequency of affect resources.

Discourse types	Affect resources	Process affect	Quality affect	Comment affect
English discourse	44 (100%)	28 (63.6%)	12 (27.3%)	4 (9.1%)
Chinese discourse	12 (100%)	12 (100%)	0 (0%)	0 (0%)

To ascertain whether there exists a disparity in the utilization of affect resources between English and Chinese discourses, a differential analysis has been conducted from three dimensions: process, quality, and comment. The findings of this analysis are presented in Table 5.

**Table 5:** Difference analysis of affect resources.

Source	SS	df	MS	*F*	*p*-value	F crit	η^2^
Amount	29	39	0.74359	1.124031	0.358433	1.704465	
Amount	3.2	1	3.2	4.837209	0.033847	4.091279	0.0551
Amount	25.8	39	0.661538				
Amount	58	79					
Amount	9.2	39	0.235897	1	0.5	1.704465	
Amount	1.8	1	1.8	7.630435	0.008709	4.091279	0.0891
Amount	9.2	39	0.235897				
Amount	20.2	79					
Amount	1.8	39	0.046154	1	0.5	1.704465	
Amount	0.2	1	0.2	4.333333	0.043984	4.091279	0.0526
Amount	1.8	39	0.046154				
Amount	3.8	79					

The results presented in Table 5 (Column 1) concerning process affect reveal that there is a significant difference between English and Chinese discourses. Specifically, the *F* value stands at 4.837209, the F critical value is 4.091279, with F exceeding F crit, the *p*-value is 0.033847, which is less than 0.05, and η^2^ value is 0.0551, which is less than 0.06. As per Table 4, in terms of frequency, the frequency of process affect in English and Chinese discourses is 63.6 and 100%, respectively. This indicates that the frequency of process affect in Chinese discourse is higher than that in English discourse, and all the affect resources in Chinese discourse are of the process affect type.

Furthermore, the outcomes in Table 5 (Column 2) for quality affect demonstrate that the difference between English and Chinese discourses is extremely significant. The *F* value is 7.630435, the F critical value is 4.091279, with F being greater than F crit, the *p*-value is 0.008709, which is less than 0.01, and η^2^ value is 0.0891, which is greater than 0.06. Regarding frequency, as illustrated in Table 4, the frequency of quality affect in English and Chinese discourses is 27.3 and 0%, respectively. This clearly shows that the frequency of quality affect in English discourse is higher than that in Chinese discourse, and there is an absence of quality affect in Chinese discourse.

Subsequently, the results in Table 5 (Column 3) for comment affect indicate that there is a significant difference between English and Chinese discourses. The *F* value is 4.333333, the F critical value is 4.091279, with F being greater than F crit, the *p*-value is 0.043984, which is less than 0.05, and η^2^ value is 0.0526, which is greater than 0.01. Additionally, as shown in Table 4, the frequency of comment affect in English discourse is 9.1%, while it is 0% in Chinese discourse, signifying the lack of comment affect in Chinese discourse.

Based on the above data analysis, it is evident that English discourse places greater emphasis on affect resources compared to Chinese discourse. From the perspective of emotional expression characteristics, English university publicity discourses center on building “simulated interpersonal interaction” with readers through “situational emotional projection”: they often use three types of affect resources—process affect, quality affect and comment affect; the combined use of these resources breaks the one-way information dissemination model of publicity discourses, simulates a face-to-face conversational feel, incorporates readers into the university’s “emotional community,” bridges the institutional-individual gap, and aligns with the personalized, humanized communication needs of core audiences like international students. In contrast, Chinese university publicity discourses have the core goal of “conveying credibility through authority construction,” resulting in “high restraint and functional simplification” in affect resource use—100% of their affect resources are process affect with an extremely low overall proportion, a choice consistent with their official discourse attribute; in expression strategies, their emotional transmission focuses on institutional stance rather than individual feelings (e.g., “to satisfy the Party and the country” ties university development goals to national strategic needs to highlight value and reliability), replacing emotional resonance with authoritative endorsement; targeting audiences like domestic examinees who care more about the university’s compliance, strength, and social contributions, these discourses adopt an objective, formal tone, prove strength by listing hard indicators such as national-level talent numbers and discipline coverage (e.g., elaborating on “high-level teachers with national outstanding contributions”), strengthen authority, help readers perceive the university as meeting national standards with solid strength, meet audience expectations for official information rigor and credibility, and ultimately shape a formal, objective, and reliable institutional image. The relevant extracts are as follows:

1. 在建设世界一流大学的进程中, XX大学将努力做到在关心国家命运与国家战略上有所作为, 让党和国家满意。[In the process of building a world-class university, XX University will strive to make a difference in caring for the destiny of the country and serving the national strategy, so as to satisfy the Party and the country] (process affect)
2. The University of XX – an inspiring place of learning and scholarship that transforms lives through: …… (quality affect)
3. We are proud of our award-winning campuses, both at home and abroad, and continually invest in the University’s grounds, buildings and facilities. (process affect)
4. In spring 2008, an exciting new chapter of XX history was launched as the Board of Trustees enthusiastically endorsed plans for a university-wide planning process…… (Comment affect)

In extract (1), the subject of perception (the sensor) is “党和国家[*the Party and the country*],” and the object of perception(phenomenon) is the behavior of “XX大学,” and the affect expressed is “满意[*satisfy*].” This sentence expresses the psychological process affect, which belongs to process affect. In extract (2), the adjective “*Inspiring*” is used to express the quality of “*place*,” that is, the University of XX is a place where people feel inspired. Extract (3) belongs to the process affect, where “we” is the subject of perception and “*campus*” is the phenomenon. Extract (4) is comment affect. The behavior of “*the Board of Trustees endorsed plans for a university-wide planning process……*” is commented.

Although English discourse tends to place greater emphasis on emotional rendering, affect resources are utilized less frequently in both English and Chinese publicity discourses. Quirk et al. (1985) argue that “discourse is semantically and pragmatically consistent with its actual context in the real world,” and Brown and Yule (2000) suggest that “discourse serves as a written record of communicative behavior.” Consequently, it is evident that the communicative intention and context significantly influence the nature of discourse. University publicity discourse, as an official form of university propaganda, is to provide society with a clearer and more accurate understanding of the university, thereby enhancing its engagement with the broader community (New Mark 1988). In the context of the objectivity requirement, the frequency of the utilization of affect resources inevitably declines. This holds true for both English and Chinese external publicity discourses. As Li and Jiang (2017) pointed out in their research on academic discourses, “The frequency of affect resource utilization in both Chinese and English is relatively low, and there are no significant disparities in terms of their distribution and realization methods.” What’s more, Zhao (2024) explored objective means such as lexis and grammar that realize attitudinal meanings in academic discourse from a cross-linguistic perspective, investigated the delicate differences between these two means, and provided insights for research and teaching in languages for specific purposes (LSP), thereby indirectly reflecting the objectivity of academic discourse.

However, in contrast to the research findings of academic discourses (Li and Jiang, 2017; Zhao, 2024) within the realm of university publicity discourses, English discourses exhibit a more pronounced emphasis on emotional expression compared to their Chinese counterparts. As is evident from the research findings presented above, English discourses demonstrate a higher frequency of emotional resource utilization, accompanied by a rich array of realization means. Conversely, Chinese discourses manifest a relatively lower frequency of emotional resource use, with a more limited and singular set of realization methods. In this respect, there is a notable convergence with the business discourses investigated by Xu and Xia (2013). Additionally, both English and Chinese discourses in these two types (university external publicity and business) possess a lower degree of objectivity when compared to academic discourses. This phenomenon is inherently determined by the unique nature of the discourses themselves and their specific communicative intentions.

### 4.3 Judgement resources

Martin and Rose (2003) contend that the resources employed for assessing personalities are referred to as judgment resources. Judgment can be categorized into two dimensions: social esteem and social sanction. Both social esteem and social sanction are further sub-divided into positive and negative aspects. In this context, positive qualities or behaviors are worthy of praise, while negative ones are subject to moral condemnation and legal criticism. Given the inherent characteristics of publicity discourse, both English and Chinese discourses predominantly feature positive comments. The following is a detailed analysis:

To determine whether there exists a disparity in the utilization of judgment resources, a differential analysis has been conducted from the two perspectives of social esteem and social sanction. The findings of this analysis are presented in Table 6.

**Table 6:** Difference analysis of judgement resources.

Source	SS	df	MS	*F*	*p*-value	F crit	η^2^
Row	680.8	39	17.45641	1.235123	0.256374	1.704465	
Column 1	57.8	1	57.8	4.089623	0.050044	4.091279	0.0448
Deviation	551.2	39	14.13333				
Amount	1289.8	79					
Row	141.2	39	3.620513	1.06006	0.428205	1.704465	
Column 2	12.8	1	12.8	3.747748	0.060151	4.091279	0.0445
Deviation	133.2	39	3.415385				
Amount	287.2	79					

The results presented in Table 6 (Column 1) regarding social esteem indicate that the difference between English and Chinese resources is negligible. This is evidenced by the fact that the *F* value is 4.089623, the F critical value is 4.091279, with F being less than F crit, the *p*-value is 0.050044, which is greater than 0.05, and η^2^ value is 0.0448, which is less than 0.06. Similarly, the findings in Table 6 (Column 2) for social sanction reveal that the difference between English and Chinese resources is also insignificant. The *F* value stands at 3.747748, the F critical value is 4.091279, with F being less than F crit, the *p*-value is 0.60151, which exceeds 0.05, and η^2^ value is 0.0445, which is less than 0.06.

From the data analysis, the difference is insignificant in the use of judgement resources in English and Chinese publicity discourses, indicating that both attach importance to the evaluation and judgment of school agents. The frequency of social esteem is higher than that of social sanction (as depicted in Table 7) in both English and Chinese discourses. Nevertheless, there is a difference in the emphasis placed on social sanction. Specifically, the Chinese discourse places a greater emphasis on compliance with national policies and laws, whereas the English discourse focuses more on the constraints imposed by moral rules. According to the Tripartite Theory of Morality (Shweder et al., 1997), the differences exist in the ethical foundations of the legal-moral relationship between China and the West. Chinese culture centers on the “community dimension,” emphasizing social harmony and collective responsibility, which leads to a high degree of integration between law and morality at the level of “obligations.” For instance, the Civil Code transforms moral duties (such as supporting parents and honesty) into mandatory norms, forming a constraint model of “legalization of morality.” Western culture places greater emphasis on the “autonomy dimension,” emphasizing individual freedom of choice and equality of rights. Moral values (such as pluralistic inclusion and equality) often precede law and drive legal reforms. This“difference in legal-moral constraints between China and the West”is reflected in the publicity discourse of university, where English discourse tends to emphasize moral constraints while Chinese discourse prioritizes legal constraints. The relevant extracts are presented as follows:

1. XX大学为民族的振兴和解放、国家的建设和发展、社会的文明和进步做出了不可替代的贡献。[XX University has made irreplaceable contributions to the rejuvenation and liberation of the nation, the construction and development of the country, and the civilization and progress of society] (social esteem)
2. 学校始终坚持马克思主义指导思想, 坚决贯彻党的教育方针, 模范执行党委领导下的校长负责制。[The university has always adhered to the Marxism guiding ideology, resolutely carried out the Party’s educational policy, and set an example of carrying out the president responsibility system under the leadership of the Party Committee] (social sanction)
3. *The title of University Professor was created in 1935 to honor individuals whose groundbreaking work crosses the boundaries of multiple disciplines, allowing them to pursue research at any of XX’s Schools* (social esteem).
4. *Being committed to excellence, enterprise and social responsibility* (social sanction).

**Table 7:** Quantity and frequency of judgement resources.

Discourse types	Judgement resources	Social esteem	Social sanction
English Discourse	264 (100%)	224 (84.8%)	40 (15.2%)
Chinese Discourse	364 (100%)	292 (80.2%)	72 (19.8%)

In extract (5), the contribution of “XX大学 [*XX university*]” is unique and outstanding. The achievements made by “XX大学” are very impressive and pleasant, belonging to the category of social esteem. In extract (6), what the university adheres to and implements is the Party’s educational policy, and the university is run under the guidance of Marxism, which belongs to the scope of legal provisions. If the university violates the law, it will be subject to legal sanctions. Therefore, the extract belongs to the scope of social sanction. In extract (7), doing groundbreaking work can endow you an honor to take a professorship, which is pleasant and belongs to social esteem. In extract (8), the university’s commitment to virtue, career and responsibility belongs to the category of morality and is a type of social sanction.

Moreover, in the publicity discourses of universities, the frequency of using judgment resources is higher than that of affective resources. This finding is highly consistent with the research results of scholars such as Shi (2018) and Li (2024b). Beyond the realm of publicity discourse, the utilization frequency of judgment resources consistently surpasses that of affect resources within legal, news, commercial, academic, and other discursive domains that uphold objectivity as a paramount principle (Wu and Yang, 2013; Xu and Xia, 2013; Wang and Tian, 2017; Shi, 2018; Monteiro and Ribeiro, 2020; Wang and Zhang, 2022; Arbieu et al., 2023; Xuan, 2023; Li, 2024a, 2024b; White, 2024). This phenomenon is, in fact, a fundamental and widespread trait inherent to discourses that emphasize objectivity, demonstrating its salience across diverse communicative contexts and textual genres.

### 4.4 Appreciation resources

“Appreciation, as defined by Martin and Rose (2003), refers to a set of resources utilized for evaluating the value of objects.” It can be categorized into three dimensions: reaction, composition and valuation. The subsequent content presents the quantity and frequency distributions of appreciation resources:

The following is an analysis of the differences in appreciation resources between English and Chinese discourses, conducted from the perspectives of reaction, composition, and valuation, with the aim of determining whether there are disparities in the utilization of these appreciation resources. The findings of this analysis are presented in Table 8.

**Table 8:** Difference analysis of appreciation resources.

Source	SS	df	MS	*F*	*p*-value	F crit	η^2^
Row	41.55	39	1.065385	1.300469	0.207834	1.704465	
Column 1	4.05	1	4.05	4.943662	0.03205	4.091279	0.0522
Deviation	31.95	39	0.819231				
Amount	77.55	79					
Row	60	39	1.538462	1.013514	0.483391	1.704465	
Column 2	64.8	1	64.8	42.68919	9.42E-08	4.091279	0.3521
Deviation	59.2	39	1.517949				
Amount	184	79					
Row	502.3875	39	12.88173	0.784383	0.774143	1.704465	
Column 3	63.0125	1	63.0125	3.836902	0.057314	4.091279	0.0522
Deviation	640.4875	39	16.42276				
Amount	1205.888	79					

9.42E-08 stands for “9.42 times 10 to the negative 8th power”.

As indicated by Table 8 (Column 1), the results demonstrate a significant difference between English and Chinese discourses in terms of reaction appreciation. The *F* value is 4.943662, the F critical value is 4.091279, with F exceeding F crit, the *p*-value is 0.03205, which is less than 0.05, and η^2^ value is 0.0522, which is greater than 0.01. The frequency of reaction appreciation is higher in the Chinese discourse compared to the English discourse, standing at 9.8 and 8.5%, respectively, (refer to Table 9).

**Table 9:** Quantity and frequency of appreciation resources.

Discourse types	Appreciation resources	Reaction appreciation	Composition appreciation	Valuation appreciation
English discourse	164	14 (8.5%)	4 (2.4%)	14 (89.1%)
Chinese discourse	325	32 (9.8%)	76 (23.4%)	217 (66.8%)

The outcome of Table 8 (Column 2) further elucidates that there is an extremely significant difference between English and Chinese discourses with respect to composition appreciation. The *F* value is 42.68919, the F critical value is 4.091279, with F being greater than F crit, the *p*-value is 9.42E-08, which is less than 0.01, and η^2^ value is 0.3521, which is greater than 0.06. The frequency of composition appreciation in the Chinese discourse is higher than that in the English discourse, specifically 23.4 and 2.4%, respectively.

Finally, the results presented in Table 8 (Column 3) reveal that there is an insignificant difference between English and Chinese discourses in relation to valuation appreciation. The *F* value is 3.836902, the F critical value is 4.091279, with F being less than F crit, the *p*-value is 0.057314, which is greater than 0.05, and η^2^ value is 0.0522, which is less than 0.06.

Based on the above analysis, differences exist in reaction appreciation and composition appreciation, and the difference in composition appreciation is particularly pronounced. Conversely, there is no difference in valuation appreciation. Regarding the frequency of appreciation resources, in the English discourse, the frequency of valuation appreciation is the highest, that of composition appreciation is the lowest, and reaction appreciation falls in between. In the Chinese discourse, the most frequently occurring resource is valuation appreciation, the least frequent is reaction appreciation, and composition appreciation is in the middle. The relevant extracts are as follows:

1. 到2020年建成国内一流、国际知名的高水平研究型大学。[By 2020, a nationally first-class and internationally renowned high-level research university will be built.] (reaction).
2. 学科点覆盖了除军事学以外的12个学科门类, 形成了综合性学科布局 [Academic field covers 12 disciplines except military science, forming a comprehensive discipline layout.] (composition).
3. 学校拥有国家级突出贡献的中青年专家、享受政府特殊津贴专家、新世纪百千万人才工程国家级人选、国家“四个一批”人才、国家“万人计划”哲学社会科学领军人才、“长江学者”青年项目、“长江学者”讲座教授等高水平师资。[The university has high-level teachers such as young and middle-aged experts with outstanding contributions at the national level, experts enjoying special government allowances, national candidates for “the New Century Ten Million Talents Project,” national “Four One Batch” talents, national “Ten Thousand People Plan” leading talents in philosophy and social sciences, “Changjiang Scholars” youth Project, and “Changjiang Scholars” chair professors.] (valuation).
4. *With deep roots in scholarship and teaching, these internationally renowned collections are fundamental to the development and continuation of many disciplines* (reaction).
5. *Offering an outstanding, broad-based, international education to talented students* (composition).
6. *These unparalleled institutions rank alongside some of the greatest museums in the world* (valuation).

In extract (9), “国内一流、国际知名[a nationally first-class and internationally renowned high-level…]” refers to the public acceptance of a university, which is the recognition and reaction of domestic and foreign people, so it belongs to reaction. In extract (10), the “综合性学科布局[a comprehensive discipline layout]” reflects that the discipline layout is comprehensive and emphasizes that the discipline structure is not single, which belongs to composition; The “高水平[high-level]” in extract (11) reflects the level and value of teachers and is classified as valuation; “*Renowned*” in extract (12) is used to describe the public acceptance of “*collections*,” which, like extract (9), belongs to reaction. In extract (13), “*broad-based”* is used to describe the basis of education provided by university, belonging to composition. In extract (14), “*greatest*” is used to describe the grade and scale of “museums,” which belongs to valuation.

Appreciation resources are also the frequently used resources in English and Chinese publicity discourse, while Chinese discourse is significantly higher than English discourse. The Chinese discourse lays more emphasis on noun phrases, which are used to introduce the teaching staff, scientific research facilities and achievements of the university, and are listed one by one, which is more detailed, while the English discourse is introduced in general and the language is concise and to the point. This is also the reason for the longer length of Chinese discourse and the shorter length of English discourse. This breaks the common thought that English discourse is long and detailed, while Chinese discourse is concise and to the point. Similarly, in terms of the length of English and Chinese discourses, university publicity discourse has the same characteristics as academic and business texts. Li and Jiang (2017), when comparing English and Chinese academic discourses, pointed out that “most Chinese prologues have longer length and more detailed content; The English prologue text is short in length and is briefly summarized”; At the same time, Xu and Xia (2013) also made the same discovery when comparing English and Chinese business texts.

## 5 Conclusion

This paper carried out ANOVA and frequency analysis of attitudinal resources in English and Chinese university publicity discourses from the three aspects of affect, judgment and appreciation, and drew the following conclusion.

Significant cross-lingual disparities emerge in the deployment of affect and appreciation resources, while judgment resources exhibit notable cross-linguistic consistency. Both English and Chinese discourses infrequently utilize affect resources; however, English discourse prominently foregrounds emotional expressions, fostering a sense of intimacy and engagement, which aligns with Western cultural values emphasizing individual emotion and interpersonal connection. In contrast, Chinese discourse prioritizes objectivity and authority, reflecting the cultural ethos of restraint and hierarchical order. Regarding judgment resources, both languages rely on them more heavily than on affect resources. English discourse predominantly appeals to moral constraints, rooted in the cultural tradition of emphasizing personal virtues and social ethics. Conversely, Chinese discourse tends to emphasize legal norms, embodying the societal focus on rule-based order. Appreciation resources are frequently employed in both linguistic contexts, yet Chinese discourse exhibits a significantly higher utilization rate, consistent with the Chinese cultural preference for rhetorical embellishment and praise. By applying the Appraisal System to cross-lingual analysis of university publicity discourses, this research has illuminated the mediating role of cultural factors in the construction of attitudinal meaning. These findings not only validate the effectiveness of the Appraisal System in cross-cultural discourse analysis but also refine its theoretical framework, expanding its application scope in cross-lingual research. This enriches the understanding of the intricate interplay among language, culture, and society, contributing novel insights to Systemic Functional Linguistics.

However, this research has certain limitations. University publicity discourse can exist either as pure text or as multimodal content integrated with images and videos. The present study focuses solely on the linguistic aspects, analyzing only the textual forms of these discourses. However, multimodal discourse analysis is equally crucial. It serves as a valuable supplement to textual analysis, offering a more intuitive way to convey the discourse content. Incorporating multimodal discourse analysis into future research on university publicity discourse will open up several exciting avenues. Firstly, future studies could focus on developing comprehensive multimodal corpora that systematically collect and catalog a wide range of publicity materials from different universities. These corpora could be annotated with detailed information about the various semiotic modes present, enabling researchers to conduct in-depth comparative analyses. For instance, a cross-cultural comparison could explore how universities in different countries use multimodal elements to communicate their unique selling points, revealing cultural differences in communication styles and values. Secondly, researchers could employ advanced computational tools and techniques to analyze multimodal data more efficiently. Machine learning algorithms, for example, could be trained to automatically detect and classify visual and audio elements in promotional videos, while eye-tracking technology could provide insights into how audiences interact with multimodal content, highlighting which elements attract the most attention and how attention is distributed over time. Thirdly, future research could investigate the impact of multimodal publicity on different target audiences. By conducting user studies and surveys, researchers could explore how prospective students, alumni, and the general public respond to various multimodal strategies. This would help universities tailor their publicity efforts more effectively, ensuring that their messages resonate with different stakeholders.

## Data Availability

The original contributions presented in the study are included in the article/supplementary material, further inquiries can be directed to the corresponding author.
